# Structural, Electromagnetic and Microwave Properties of Magnetite Extracted from Mill Scale Waste via Conventional Ball Milling and Mechanical Alloying Techniques

**DOI:** 10.3390/ma14227075

**Published:** 2021-11-22

**Authors:** Madiha Fathi Elmahaishi, Raba’ah Syahidah Azis, Ismayadi Ismail, Muhammad Syazwan Mustaffa, Zulkifly Abbas, Khamirul Amin Matori, Farah Diana Muhammad, Nor Kamilah Saat, Rodziah Nazlan, Idza Riati Ibrahim, Nor Hapishah Abdullah, Nurhidayaty Mokhtar

**Affiliations:** 1 Department of Physics, Faculty of Science, University Putra Malaysia (UPM), Serdang 43400, Selangor, Malaysia; gs57071@student.upm.edu.my (M.F.E.); za@upm.edu.my (Z.A.); khamirul@upm.edu.my (K.A.M.); farahdiana@upm.edu.my (F.D.M.); kamilah@upm.edu.my (N.K.S.); hidayaty@upm.edu.my (N.M.); 2 Materials Synthesis and Characterization Laboratory (MSCL), Institute of Nanoscience and Nanotechnology (ION2), Universiti Putra Malaysia (UPM), Serdang 43400, Selangor, Malaysia; ismayadi@upm.edu.my (I.I.); mm_syazwan@upm.edu.my (M.S.M.); 3 Faculty of Industrial Sciences, Technology Universiti Malaysia Pahang (UMP), Lebuhraya Tun Razak, Gambang 26300, Pahang, Malaysia; rodziah@ump.edu.my; 4 Centre For Pre-University Studies, Universiti Malaysia Sarawak (UNIMAS), Kota Samarahan 94300, Sarawak, Malaysia; iiriati@unimas.my; 5 Functional Devices Laboratory (FDL), Institute of Nanoscience and Nanotechnology (ION2), Universiti Putra Malaysia (UPM), Serdang 43400, Selangor, Malaysia; hapishah@upm.edu.my

**Keywords:** magnetite (Fe_3_O_4_), mill scale, conventional milling (CM), mechanical alloying milling (MM), microwave, electromagnetic properties

## Abstract

This study presents the utilization of mill scale waste, which has attracted much attention due to its high content of magnetite (Fe_3_O_4_). This work focuses on the extraction of Fe_3_O_4_ from mill scale waste via magnetic separation, and ball milling was used to fabricate a microwave absorber. The extracted magnetic powder was ground-milled using two different techniques: (i) a conventional milling technique (CM) and (ii) mechanical alloying (MM) process. The Fe_3_O_4_/CM samples were prepared by a conventional milling process using steel pot ball milling, while the Fe_3_O_4_/MM samples were prepared using a high-energy ball milling (HEBM) method. The effect of milling time on the structural, phase composition, and electromagnetic properties were examined using X-ray diffraction (XRD) and a vector network analyzer (VNA). XRD confirmed the formation of magnetite after both the magnetic separation and milling processes. The results revealed that Fe_3_O_4_ exhibited excellent microwave absorption properties because of the synergistic characteristics of its dielectric and magnetic loss. The results showed that the Fe_3_O_4_/CM particle powder had a greater absorption power (reflection loss: <−10 dB) with 99.9% absorption, a minimum reflection loss of −30.83 dB, and an effective bandwidth of 2.30 GHz for 2 mm thick samples. The results revealed the Fe_3_O_4_/MM powders had higher absorption properties, including a higher RL of −20.59 dB and a broader bandwidth of 2.43 GHz at a matching thickness of only 1 mm. The higher microwave absorption performance was attributed to the better impedance matching property caused by the porous microstructure. Furthermore, the magnetite, Fe_3_O_4_ showed superior microwave absorption characteristics because of the lower value of permittivity, which resulted in better impedance matching. This study presents a low-cost approach method by reutilizing mill scale waste to fabricate a high purity crystalline Fe_3_O_4_ with the best potential for designing magnetic nano-sized based microwave absorbers.

## 1. Introduction

Microwave absorbing materials (MAMs) are materials that can interact with electromagnetic (EM) waves and dissipate into other forms of thermal energy through their electric and magnetic losses [1,2,3]. Such materials with broadband absorption performance have attracted great interest due to their important applications in high-speed communications technology, electronic stability, health care, and national defense security [4,5,6,7,8]. Nowadays, the high environmental pollution of electromagnetic (EM) waves produced by the accelerating of variable electronic applications such as wireless communication technologies in mobile phones, remote controls, and Wi-Fi has led to serious harm to human healthcare and has affected the normal operation of electronic equipment and communications systems that operate at super high frequency ranges [9,10,11]. As a viable solution to tackle this issues, advanced electromagnetic absorptive materials have attracted considerable attention because they exhibit strong absorption, light weight, wide absorption bandwidth, reduced thickness MAMs, strong attenuation, and good impedance match [12,13,14]. In addition to their outstanding absorption capacity, these materials can maximize the electromagnetic energy entering a material and convert that energy into thermal energy or dissipate it via an interference-effect based on their dielectric or magnetic loss mechanisms, which are determined by the parameters of electromagnetism, i.e., complex permeability and permittivity during the absorbing process of an electromagnetic wave [15,16,17]. 

In the past decade, magnetic materials such as carbonyl iron, spinel ferrites, hexagonal ferrites, and various kind of ferrites have been investigated as MAMs for microwave absorbing materials [18,19,20,21]. To achieve strong magnetic dissipation, the use of magnetite (Fe_3_O_4_) extracted from mill scale waste is promising to improve EM wave absorption due to its low cost, simple production, strong adsorption capacity, easy separation, and enhanced stability [22,23]. Mill scale waste comprises a flaky surface of hot rolled steel introduced by industrial by-products that contain iron (II) oxide (FeO), iron (III) oxide (Fe_2_O_3_), and iron (II, III) oxide (Fe_3_O_4_, magnetite) [24]. A study by Nazri et al. successfully proved that industrial mill scale waste has good potential to produce a mass volume of magnetite (Fe_3_O_4_) particles with strong magnetic properties. The samples were extracted and grounded via conventional steel ball milling and high-energy ball milling (HEBM) at various milling times; consequently the MAMs were characterized [22]. Magnetite (Fe_3_O_4_) is an ideal magnetic loss absorber, as well as a hard magnetic material that has proper saturation magnetization (M_s_), high coercivity (H_c_), excellent chemical stability, low toxicity, and hetero-interfaces formed by impurities in addition to its outstanding permeability and permittivity that promote high EM wave absorption [25,26]. Based on these characterizations, numerous studies on magnetite have been conducted [27,28,29]. Because of the major promise of iron oxide nanoparticles (NPs) for future biomedical applications—as well as their magnetic properties and other intrinsic properties such as low toxicity—a review by Wei et al. examined various synthetic techniques and surface engineering methodologies for generating naked and functional iron oxide NPs with various physicochemical characteristics [30]. According to a study of Pawar et al., a high-performance electromagnetic wave absorber with high surface resistivity and enhanced attenuation constant was created in blends containing both MWNTs and rGO Fe_3_O_4_, which manifested in a total shielding effectiveness of −50.7 dB (at 18 GHz), indicating the >99.9% attenuation of the incoming microwave radiation [31]. In addition, using an electrostatic self-assembly technique, highly controlled Fe_3_O_4_–polyelectrolyte-modified polyaniline (Fe_3_O_4_–PE@PANI) hollow spherical nanocomposites were effectively produced by Zhu et al. [32] In the 0.5–15 GHz frequency range, the Fe_3_O_4_–PE@PANI nanocomposites were shown to have outstanding reflection loss abilities and large response bandwidths compared to PANI hollow spheres. As a result, the Fe_3_O_4_–PE@PANI nanocomposites show promise in microwave absorption applications. Recently, based on the influence of the ball-to-powder weight ratio on the crystal composition, ] purified palygorskite was used as a template to create palygorskite/Fe_3_O_4_ nanocomposites with the conventional wet ball-milling process [33]. The Fe_3_O_4_-to-palygorskite weight ratio was modified to 2:1, resulting in enhanced interface polarization and a maximum reflection loss (RL) of −40.41 dB at 4.80 GHz, with an effective absorption frequency that could be adjusted across 2–18 GHz by changing the composite’s thickness. Moreover, Jian et al. discovered nanoscale Fe_3_O_4_@graphene capsule (GC) composites with a minimum RL_min_ value of −32 dB at 8.76 GHz and an EAB that rose from 5.4 to 17 GHz as the sample thickness increased [34]. An adequate interface contact in a composite absorbing material is useful for increasing dielectric loss and enhancing microwave absorption performance. Meng et al. fabricated a small size composite of Fe_3_O_4_ covered with an ultra-thin carbon layer (Fe_3_O_4_/C); the prepared sample displayed the strongest reflection loss of composite NPs with an average particle size of 52 nm; this composite could reach −58.5 dB at 14.88 GHz with a thickness of 2 mm, and the corresponding effective absorption (RL ≤ −10 dB) bandwidth (EAB) was 5.63 GHz (12.37–18 GHz) [35].

Though magnetite is very attractive as an MAM, it has some limitations because of its rapid decrease in permeability at microwave frequencies, large density, impedance mismatch, and poor antioxidation [13]. To enhance the properties of magnetite and provide advantageous properties for the use of as-prepared Fe_3_O_4_ to design MAMs, one can improve the size of the particle using ball-milling techniques. Ball milling cannot reduce particle size without simultaneously introducing a variety of defects that improve dielectric relaxation. During the ball-milling process, multi-interfacial polarization is produced in magnetite, resulting in an increase in Fe_3_O_4_‘s microwave absorption performance. Additionally, the synergy impact of dielectric and magnetic losses may be tuned to provide optimum impedance matching. The results of several works showed a correlation between the ball-milling times and various properties such as crystallite and particle size, specific surface area, magnetic properties, and nanoscale porosity of the iron powder [36,37,38]. Yan et al. obtained magnetite particles (MPs) using the high-energy ball milling of oxidized nickel slag that possessed strong magnetic properties and hetero-interfaces formed by impurities. According to their results, magnetite crystals displayed outstanding microwave absorption properties [39]. At a thickness of 5 mm, the lowest reflection loss (RL) of the particles produced after 6 h of ball-milling was −34.0 dB at 16.72 GHz. The effective frequency bandwidths (RL ≤ −10 dB)) were 4.8–5.4 at 15.9–17.6 GHz, respectively. Furthermore, Liang et al. obtained Fe_3_O_4_ with different particle sizes via mechanical ball milling by controlling the milling time [38]. Their results showed that when the ball-milling time rose, the integrity of the original small spherical structure decreased. With a milling time of 2 h, Fe_3_O_4_ demonstrated great microwave absorption, with a maximum reflection loss of −21.19 dB at 4.64 GHz and a thickness of 6.55 mm. The performance of Fe_3_O_4_/Fe composites as magnetic microwave absorbers was confirmed by a study of Cheng et al. [40]. In their study, the ball-milling process had a huge influence on the morphology and electromagnetic absorption capability of these composites, where reflection loss values below −10 dB were achieved from 13.8 to 18 GHz, with a maximum reflection loss of −25.9 dB at 16.1 GHz.

Tremendous research has been conducted to develop electromagnetic wave characteristics for microwave absorbing materials to design composites loaded with different types of particles of variable size for use in electromagnetic microwave absorbing materials, in addition to investigating the effect of milling time on particle size and microwave absorption performance [38,41,42]. Because microwave absorption is highly related to particle size, ball milling was conducted to improve the EM wave characteristics of Fe_3_O_4_, as ball milling allows for the convenient adjustment of particle size [43]. The proper choice of nanoscale materials is key in achieving the multifunctional properties that are required by microwave absorption applications, and the synthesis of microwave absorber composites using polymers and magnetic filler at different scales using the simplest mechanical ball-milling method has an important place in this research. For instance, the impact of milling time on the microwave absorption properties of barium hexaferrite nanoparticles was studied by Gunanto et al. [44]. Their results demonstrated that as milling time increased, grain size dropped and maximum peak absorption rose significantly. As the powder sample was milled for 25 h, the minimum reflectivity of the absorbent sample was −20 dB at 10.9 GHz. At a frequency of roughly 11.2 GHz and a bandwidth of 1.5 GHz, the greatest reflection loss (RL) for 50 h of milling was approximately −33 dB, and the absorption peak (RL) was about −49 dB at a frequency of 10.8 GHz with bandwidth of 1.7 GHz for 75 h of milling. Furthermore, a study by Effendi et al. [18] showed that increasing the milling time by more than 1 h was beneficial for enhancing the microwave absorption characterizations of strontium lanthanum ferrite compounds because it caused the sample to agglomerate, causing the crystal size to increase, the Sr_0.5_La_0.5_Fe_12_O_19_ crystal phase and saturation magnetization (Ms) values to decrease, and the microwave absorption ability to decrease. However, according to previous literature, the influence of variations in milling time on the microwave absorbing properties of magnetite Fe_3_O_4_ (gained from mill scale waste and processed via the magnetic separation technique) has not been reported so far. As part of intensive research to study the microwave absorbing characteristics of magnetite, the authors of this paper aimed to study the effect of different particle sizes and various milling times on Fe_3_O_4_ from mill scale waste products.

## 2. Materials and Methods

### 2.1. Magnetic Separation from Mill Scale Waste

The raw mill scale waste was obtained from steel factories in Malaysia. The mill scale weighed about 100 g, and it was ground and crushed using steel ball milling for several hours to obtain a fine powder. The milled powder was purified by using the magnetic separation technique (MST) to distinguish the magnetic compound Fe_3_O_4_ from non-magnetic particles (wood, stone, sand, and the elements C, O, N, P, Si, and Mn). The magnetic separation routes were carried out similarly to the previous report by Azis et al. [23]. The powder was placed in a glass tube with deionized water in the presence of an external magnetic field of 1 T. The impurities or non-magnetic particles that settled at the top of the tube due to their low density were removed, as were the impurities that were deposited at the bottom due to their high density. The magnetic powder was then filtered and placed on flask to dry in an oven overnight at 65 °C. The magnetic particles then were again separated in form the iron oxide (FeO and Fe_3_O_4_) using the Curie temperature separation technique (CTST) with a magnetic field of 1 T. In the CTST process, the powder was put into the tube with deionized water at 90–100 °C and vertically shaken along the magnetic field. We separated the magnetic particles by varying the Curie temperature of the FeO and Fe_3_O_4_ particles. The weak ferromagnetic particles dropped to the bottom of the tube and strong magnetic particles with high Curie temperature were attracted to the magnet. Here, we expected that at a finite temperature, the vector direction of the magnetic moment of each particle was randomly directed or became magnetic. Thus, we describe these particles as FeO and wustite with a low Curie point of 78 °C. Then, the magnetic particles at the center of the glass tube were filtered and dried in the oven.

### 2.2. Preparation of Fe_3_O_4_ Powders

#### 2.2.1. Conventional Ball-Milling Technique

The raw mill scale waste was obtained from steel factories in Malaysia. The mill scale weighed about 200 g, and it was ground and crushed using conventional steel ball milling for 1, 4, and 8 days to obtain powder samples.

#### 2.2.2. High-Energy Ball-Milling Technique

**Figure 1:**
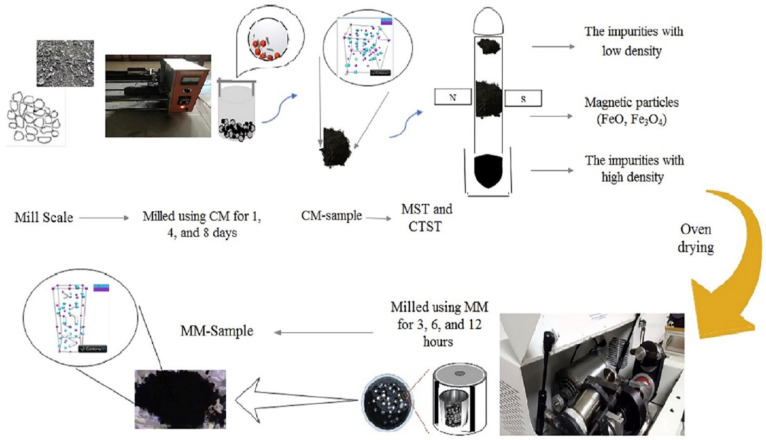
Schematic design of the preparation process of the samples using the conventional milling method (CM) and mechanical alloying (MM) process to obtain powder particles with different sizes.

The powder mixtures were further milled using mechanical alloying with a high-energy ball milling (HEBM) SPEX8000D mill (Metuchen, SpexCertPrep, NJ, USA) in a hardened steel vial together with grinding balls with a diameter of 12 mm each. We used milling times of 3, 6, and 12 h and a ball-to-powder weight ratio (BPR) ratio of 10:1, with a fixed rotating speed of 1450 rotations per minute (rpm), to obtain MM-powder (see Figure 1).

### 2.3. Composite Preparation and Microwave Properties Measurement

**Figure 2:**
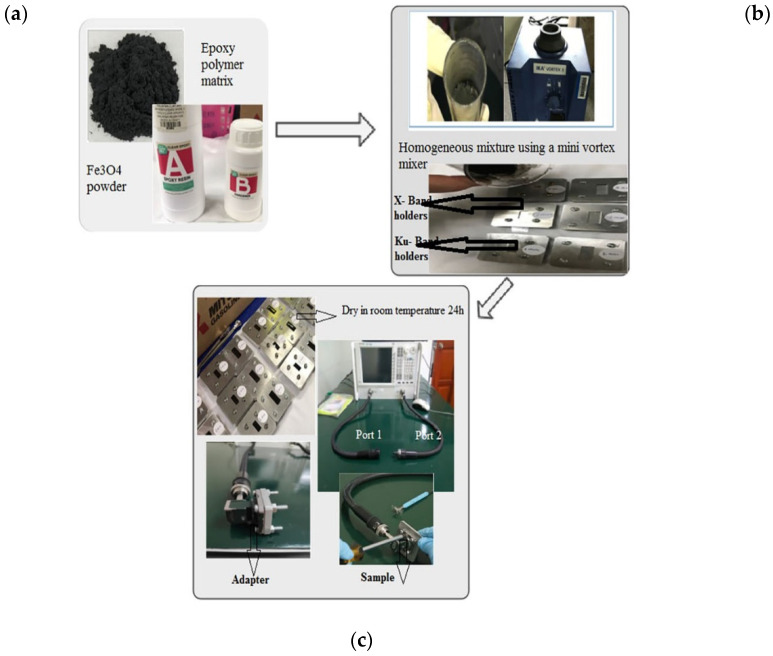
(**a**) Ferrite powder and epoxy polymer matrix; (**b**) the mixing process and the pouring of a nascent mixture into holder; (**c**) the drying of the composite followed by use of a vector network analyzer connected to the sample holder with an adapter and metal short.

The composite samples were prepared for electromagnetic microwave absorption measurements. The Fe_3_O_4_ powder was mixed with epoxy resin as a matrix. The composite samples were prepared by mixing the Fe_3_O_4_ powder samples in an epoxy polymer matrix at a weight ratio of 70:30, and then the mixture was homogeneously mixed by using a mini vortex mixer for 30 min at a speed of 3000 rpm. The mixtures were poured in the standard waveguide rectangular mold with inner dimensions of 23 × 10 mm (X band) and 15 × 7 mm (Ku band), with fixed thicknesses of 1, 2 and 3 mm. The samples were dried for 24 h at room temperature (see Figure 2). The variations of complex permeability, complex permittivity, and reflection loss (RL) with the frequency of the composite samples were analyzed using a PNA network analyzer N5227A via the transmission reflection line method (TRL). The composites specimens were measured in the 8–12 GHz (X band) and 12–18 GHz (Ku band) frequency ranges.

### 2.4. Characterization of the Samples

The crystallinity and phase compositions of the samples were examined using X-ray diffraction (XRD) (Philips X’pert Diffractometer model 7602 EA Almelo, Akishima-shi, Tokyo 196-8666, Japan). The surface microstructures of the CM and MM Fe_3_O_4_ samples were analyzed using an FEI Nova Nano 230 Field Emission Scanning Electron Microscope (FESEM Kensington, Sydney, Australia) equipped with an energy-dispersive X-ray (EDX) system. The complex permeability for prepared samples was measured using a vector network analyzer VNA (PAN-X N5244A) in the range of 8–18 GHz (X and Ku bands), and microwave absorbing characteristics were calculated according to the transmission line theory. 

In this study, the variation of the average crystallite size is represented by the broadening of FWHM. The strain (ε) and the average crystallite size (DXRD), in the relationship with FWHM, were estimated using the Williamson–Hall method (Equation (1)) [39,45]:

(1) $$\beta_{hkl}\cos\theta_{hkl}=\frac{0.9\lambda }{D}+4\varepsilon \cdot sin\theta_{hkl}$$

where λ is the CuKα radiation wavelength (1.5406 Å), *β* is the full width at half maximum intensity (FWHM), *hkl* represents the Miller index, and θ is the Bragg angle (^o^).

Bragg’s law with Nelson–Riley correction was used to derive the lattice constant, a (Å) (Equation (2)) [46,47,48]:

(2) $$a=\frac{\lambda \sqrt{h^{2}+k^{2}+l^{2}}}{2sin\theta }$$

where is λ the CuKα radiation wavelength (1.5406 Å), *hkl* is the Miller index, and θ is the Bragg angle (^o^).

## 3. Results and Discussion

### 3.1. Structural Analysis

**Figure 3:**
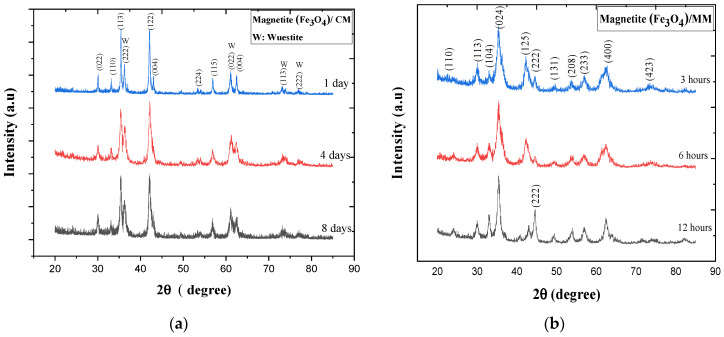
XRD spectra of Fe_3_O_4_ samples at different milling times: (**a**) Fe_3_O_4_/CM with milling times of 1, 4, and 8 days; (**b**) Fe_3_O_4_/MM obtained with milling times of 3, 6, and 12 h.

Figure 3 presents the analyzing of the effect of ball-milling time on the structures of the Fe_3_O_4_/CM and Fe_3_O_4_/MM samples using Philips X’pert diffractometer. Figure 3a shows the XRD characterization of Fe_3_O_4_/CM at different ball-milling times. The diffraction patterns of the sample after 1 day of milling time showed that the magnetite phase was the principal phase of the powder, along with other phases of wustite (FeO). However, after 4 and 8 days of milling, wustite FeO peaks disappeared and a single phase of Fe_3_O_4_ was observed. Obviously, the diffraction peaks at 2θ of (°) = 30.12°, 35.37°, 43.03°, 53.39°, 56.91°, 62.49°, 73.44°, and 77.03, respectively, corresponded to the (220), (113), (111), (004), (224), (115), (044), and (335) planes and were assigned to the face-centered cubic Fe_3_O_4_ (JCPDS-98-004-1424). All peaks agreed with the results of earlier studies [49,50,51,52]. The intensity of the high peak corresponding to the (113) plane was consistent with the literature. Furthermore, the intensity of the Fe_3_O_4_ diffraction peaks dropped as the CM ball-milling time increased. This demonstrated that as the milling time rose, the milling energy increased and the Fe_3_O_4_ particles broke up. Therefore, the grain size shrank and the substance was further refined. No obvious XRD peaks originating from the impurities were found, which means that magnetite at the microscale was successfully synthesized from mill scale waste with high purity.

The diffraction peaks of Fe_3_O_4_/MM shown in Figure 3b had the same pattern and verify that there no other phase transformation occurred after milling, as there is no peak other than that of the iron oxide phase. This illustrates that the variation of milling time did not affect the phase composition of the Fe_3_O_4_/MM particles. The peaks for the three different milling times (3, 6, and 12 h) were almost the same. As seen from the patterns, the XRD peaks of all samples after HEBM mechanical alloying in Figure 3b corresponded to the diffraction pattern characteristic of the magnetite structure (JCPDS: 98-007-7864) observed at the 2θ (°) of 21.07°, 26.54°, 37.17°, 43.07°, 53.82°, 57.02°, and 62.57° representing the (110), (113), (024), (125), (208), (233), and (400) crystalline planes of magnetite, respectively, which matched the Fe_3_O_4_ model data (JCPDS: 98-005-9302) and corresponded with the literature [53]. It is clear that all of the diffraction peaks were sharp, indicating that the sample was crystalline in nature and did not go through an amorphous phase throughout the milling process. The sharp and strong peaks of XRD in all samples indicated that the prepared samples had a small crystallite size. 

The cell volume (Vcell) and structural parameters of lattice constants of a, b, and c obtained from XRD data analysis are listed in Table 1. It can be seen that the lattice parameters did not show any considerable changes with varying milling times.

**Figure 4:**
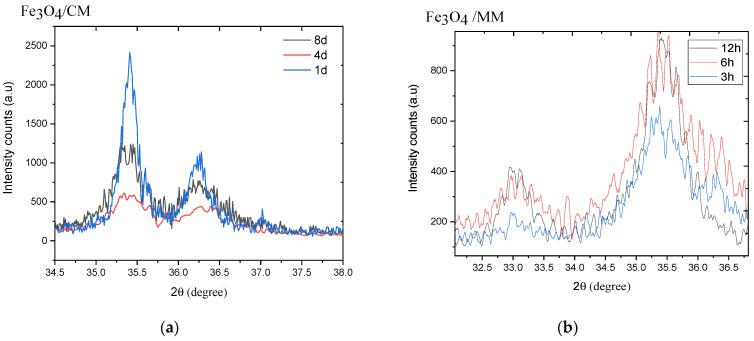
The relationship between the XRD peak intensity of the Fe_3_O_4_ samples and different milling times: (**a**) Fe_3_O_4_/CM with milling times of 1, 4, and 8 days; (**b**) Fe_3_O_4_/MM with milling times of 3, 6, and 12 h.

The XRD patterns of Fe_3_O_4_/CM powders were recorded with increases in milling time to explore changes in the crystal structures of the samples during the milling process (1, 4, and 8 days). The XRD patterns showed that the sample with 1 day of milling time had a narrow scattering peak with a relatively high intensity at 35.4° (Figure 4a); with increasing milling times, the intensity of the peak slightly decreased, the peak size of the peak slight increased, and the width rapidly increased. This indicates a decrease in the crystallinity of the ball-milled particles. Furthermore, the XRD patterns revealed that the diffraction peaks of the 3 h Fe_3_O_4_/MM became shorter and wider before gradually increasing in length and size along with the increase in the milling time to 6 h, as shown in Figure 4b. However, the diffraction peaks did not change much when the milling time was increased to 12 h.

The crystallinity percentages of varying milling times for Fe_3_O_4_/CM and Fe_3_O_4_/MM were calculated from XRD based on Equation (3) [54]:

(3) $$The\;crystallinity\;\%=\frac{Areas\;of\;crystalline\;peaks\;}{Areas\;of\;all\;crystalline\;peaks\;\left(crystalline+amorphous\right)}\times 100$$

The analysis of XRD diffraction peaks revealed that samples of that were milled for 1 and 8 days had a higher crystallinity percentages than the sample milled for 4 days. In the crystallization stage of the 1 d Fe_3_O_4_, although amorphous phases appeared, crystalline-state phases still comprised much content; the crystallinity was approximately 79%. For the 8 d Fe_3_O_4_, there were no completely amorphous phases and the content of the crystalline-state phases increased to 80.16%, as tabulated in Table 1. There were no completely amorphous phases for the samples prepared via HEBM, and the phases were complete crystalline-state phases. The CM and MM samples both exhibited increased crystallinity with increases in milling time, but the crystallinity percentages showed that the fluctuation of crystallinity was very small after the samples were subjected to various milling times.

**Figure 5:**
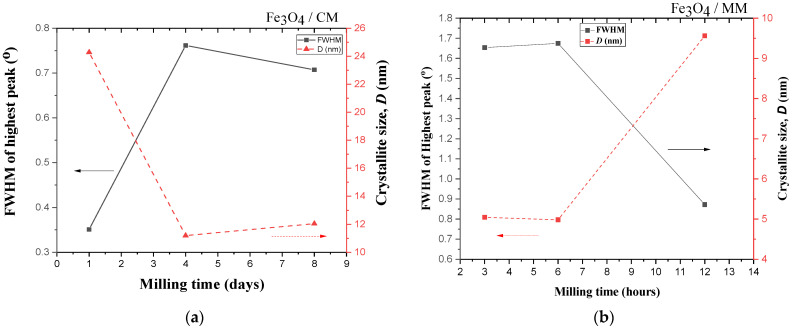
The full-width at half-maximum (FWHM) and crystallite size of: (**a**) Fe_3_O_4_/CM milling for 1, 4, and 8 days; (**b**) Fe_3_O_4_/MM milling for 3, 6, and 12 h.

Figure 5 shows the production of crystalline magnetite was determined by the widening of XRD peaks as the milling time increased. However, we also observed that as the milling time increased, the XRD peak intensities decreased and the width of the peaks broadened. This also indicated a decrease in the particle size of the samples [55]. In addition, the strain created during the milling process also resulted in a drop in peak intensity and the widening of diffraction peaks as the particle size decreased. To additionally reveal changes in the microscopic crystal properties of the magnetite powder with increases in the milling time, the relationship between FWHM and crystallite sizes (calculated by Equation (1)) is shown in Figure 5. Figure 5 shows the influence of milling time on the FHWM and crystallite for Fe_3_O_4_/CM (Figure 5a) and Fe_3_O_4_/MM (Figure 5b). The analysis showed that the increase in milling times from 1 to 4 and 8 days for the Fe_3_O_4_/CM samples and from 3 to 6 and 12 h for the Fe_3_O_4_/MM samples led to changes in the FWHM and the average crystallite size of Fe_3_O_4_ (Table 2). For the CM method, the increases in milling time from 1 to 4 and 8 days led to decreases in the average crystallite size from 24 to 11 and 12 nm, respectively; these results indicate that decreases in the crystallite size of Fe_3_O_4_/CM are typically reflected by the broadening of the FWHM. For the MM method, increasing the milling time from 3 to 6 and 12 h led to increases in the average crystallite size from 5.01 to 4.98 and 9.56 nm, respectively; meanwhile, the FWHM showed notable widening, and the diffraction peak intensities of the 12 h Fe_3_O_4_/MM were clearly attenuated, which was linked to the distortion of grain refinement and lattice deformation caused by ball-milling action. In the MM method, powder particles are restricted between highly kinetic colliding balls and the inner surface of the vial, leading to the repeated deformation, rewilding, and fragmentation of premixed powders, as well as the production of fine and dispersed particles in the grain-refined matrix [56]. During a long milling process, the increase of cold welding process leads to an increase in average crystallite size of a sample [57]. Furthermore, extended milling times for our samples caused congestion that eventually turned into an agglomeration, and the crystallite size grew larger. This phenomena were consistent with Zhang et al. previously’ published findings [45].

### 3.2. Microwave Absorbing Properties of Fe_3_O_4_

In accordance transmission line theory, the EM wave absorption properties represented by the reflection losses (RLs) of the Fe_3_O_4_/CM and Fe_3_O_4_/MM particles were measured at room temperature in the frequency range of 8–18 GHz (X and Ku bands) using a vector network analyzer. *z*_0_ = *z_in_* = 1 represents ideal absorbing properties when a material enables an EM wave to effortlessly pass through and be totally absorbed by itself; Z_0_ is the impedance of free space and *z_in_* is the input impedance at the free space-absorbing materials interface. RL refers to the microwave absorption performance of samples, which was determined using complex relative permittivity and permeability spectra that change with different frequencies and thicknesses as follows [58]:

(4) $$RL\;\left(\mathrm{dB}\right)=20\log\frac{\left(z_{in}-1\right)}{z_{in}+1}$$

where $z_{in}$ is the normalized input impedance obtained from Equation (5):

(5) $$z_{in}=z_{0\;}\sqrt{\frac{\mu_{r}}{\varepsilon_{r}}}\tan h[j\left(\frac{2\pi ft}{c}\right)\sqrt{\mu_{r}\varepsilon_{r}}$$

$$z_{0}=\sqrt{\frac{\mu_{0}}{\varepsilon_{0}}}$$

where $z_{0}$ and $z_{in}$ are the normalized input and free space characteristic impedance, respectively; *ε*_0_ and *µ*_0_ are the permittivity and permeability of free space, respectively; $\varepsilon_{r}$ and $\mu_{r}$ are the complex permittivity and permeability of the composite, respectively; *h* is the Planck constant; j = √(−1), c is the velocity of electromagnetic waves in free space; t is the thickness of the material; and f is the microwave frequency. Generally, having RL values of less than −10 and −20 dB indicates more that than 90 and 99%, respectively, of the introduced EM wave has been absorbed [59]. The bandwidth of the absorber represents the frequency band corresponding to the reflection loss (R < −10 dB) at a fixed absorbing thickness.

Magnetite (Fe_3_O_4_) is a mineral with an inverse cubic spinel structure. Magnetite crystallizes with a unit cell that comprises iron ions that form tetrahedral and octahedral structures, as well as oxygen ions in a close-packed (face-centered cubic) FCC structure [59]. Iron ions have a chemical formula (AB_2_O_4_), where A is the tetrahedral position that expresses a divalent metal (Fe^3+^) and B is the octahedral position that represents a trivalent metal (eight Fe^2+^ and eight Fe^3+^ ions). Additionally, if all the divalent metal ions occupy the B site, then the inverse spinel structure is formulated, where half a portion of the trivalent metal ions occupies the B site and half of the trivalent metal ions occupy the A site [60]. The XRD patterns show that magnetite (Fe_3_O_4_) has an inverse spinel cubic structure and is an important magnetic absorbent. Half of Fe^3+^ occupies the tetrahedral position (position A) in the structure of Fe_3_O_4_ (AB_2_O_4_), and Fe^2+^ and the other half of Fe^3+^ are present in the octahedral position (position B). Electrons can move rapidly between Fe^2+^ and Fe^3+^ in the B position, so this property contributes to the superb microwave absorption performance of Fe_3_O_4_. In addition, because the frequency is sufficiently high, Fe^2+^ can easily polarize, resulting in significant EM wave loss. Moreover, Fe_3_O_4_’s natural resonance and eddy current loss also considerably contribute to electromagnetic wave absorption [61]. 

**Figure 6:**
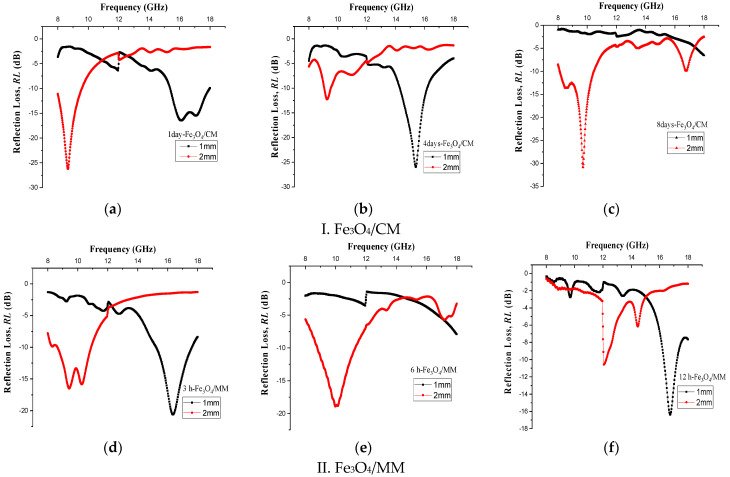
(**I**) Reflection loss curve (RL) for Fe_3_O_4_/CM with milling times of (**a**) 1 day, (**b**) 4 days, and (**c**) 8 days at thicknesses of 1 and 2 mm; (**II**) reflection loss curve (RL) for Fe_3_O_4_/MM with milling times of (**d**) 3 h, (**e**) 6 h, and (**f**) 12 h at thicknesses of 1 and 2 mm.

Figure 6 presents the frequency- and thickness-dependent reflection loss (RL) curves for all prepared Fe_3_O_4_ samples, and the value of maximum reflection loss with respect to the frequency for each milling time is tabulated in Table 3. The raw absorbent materials used in this study were magnetic materials that were easily created at low cost from mill scale waste; they had strong magnetic properties that led to the expansion of the absorption frequency, and they had large magnetic saturation values. RL curves were plotted for optimal match thickness, supplying each frequency band its maximum effective bandwidth (bandwidth at <−10 dB). It can be seen that the frequency and amplitude of the minimum reflection loss RL (maximum absorption) varied with the milling and thicknesses of the absorber. In order to create thin, light, and wide absorbent materials, the material thickness should not be too large. The results showed that the best matching thicknesses of magnetite samples were 1 and 2 mm, so 1 and 2 mm were selected as the standard thicknesses to investigate the effect of milling time on the microwave absorption properties. On the other hand, weak absorption was gained by the CM and MM Fe_3_O_4_ powders in the X and Ku bands at the high matching thicknesses of 3 mm.

The microwave absorbing properties of a good absorbent material can be determined when an absorber exhibits very high-performance reflection loss. Among all the prepared magnetite samples, the sample milled for 8 days presented the highest absorption peaks of the minimum RL < −30.83 dB, with absorption percentages of ~99.9% at 9.72 GHz at a matching thickness 2 mm. We observed that in the test frequency range, all three samples performed well in terms of microwave absorption. Meanwhile, as the milling time increased, the optimum RL of the Fe_3_O_4_/CM samples shifted from low to high frequency bands. Specifically, for samples milled for 1 day, the minimum RL was −26.21 dB at 8.66 GHz, and for samples milled for 4 days, it was −25.96 dB at 15.39 GHz; these values were achieved at frequency bandwidths of 1.76 and 1.86 GHz and 2.0 and 1.0 mm thicknesses, respectively. Their performance corresponded to a wave absorption of above 90% EM. For samples with a longer milling times, the broad effective bandwidth (RL < −10 dB) of greater reflection loss peaks was a bit wider, and the thickness was a little thinner. Overall, we created samples with an RL exceeding −10 dB in a wide frequency range of 1.76–18 GHz for a milling time of 1 day, 1.86–18 GHz for 4 days of milling time, as well as a sample that milled for 8 days with an RL exceeding −20 dB in the frequency range of 2.30–18 GHz. This findings illustrated that the conventional milling technique was an effective, simple, and straightforward approach for producing large crystalline powders, with the ability to acquire vast amounts of materials with modified properties. Particle size was shown to have great influence on resonance frequency, as larger and smaller particle sizes were found at lower and higher frequency ranges, respectively [62]. Consequently, the microwave absorption curves of Fe_3_O_4_/CM displayed enhanced RL values and the absorption peaks moved to lower frequencies after the sample thickness was increased to 2 mm, and these samples were expected to have large particle sizes.

According to the reflection loss spectrum, Fe_3_O_4_/MM with a smaller particle size (after 3 h of milling) had the best microwave absorption performance of −20.59 dB at a high frequency band (16.35 GHz) and thickness of 1 mm. This outperformed the competition in terms of peak absorption height and bandwidth. Hence, ball-milling operations can improve RL values and effectively expand bandwidth. The RL value for the sample milled for 3 h was close to that of the sample milled for 6 h, though it had a smaller particle size and higher defect density [39]. This finding was consistent with XRD characterization data, which indicated that samples milled for 3 and 6 h yielded the smallest crystal sizes. However, because the particles in our sample prepared via the MM technique were at a small scale, new absorption mechanisms emerged and the microwave energy was effectively absorbed. In other words, the Fe_3_O_4_ particles’ exceptional microwave absorption property is dictated by not only Fe_3_O_4_’s natural character but also the influence of its small size [63]. It was found that Fe_3_O_4_ created with small magnetic particle sizes cannot support more than one domain due to its small size and its ferromagnetic resonance absorbing more energy, thus revealing that decreasing particle size is beneficial for improving microwave absorption. This factor resulted in the optimum microwave absorption performance for Fe_3_O_4_/MM samples milled for 3 and 6 h. In other words, 3 and 6 h are the best milling times for achieving a high microwave absorption capability. Increasing the milling time to 12 h led the sample to agglomerate, causing the crystal size to increase, the Fe_3_O_4_ crystal phase to drop, and the microwave absorption performance to eventually decline. Furthermore, the high microwave absorption performance caused by such variations in milling time may be associated with the crystallization percentages and magnetic loss mechanism of magnetite [4]. Regarding XRD peaks, the content of the amorphous phase was very small and that of the crystalline state was obviously high at all milling times. Despite that, we observed that the fluctuation of crystallinity did not influence the reflection loss.

The high microwave absorption performance of the CM samples was compared to that of the MM samples. A summary of this comparison is included in Table 4, which shows the absorption percentages for all samples. Based on the principles of the mechanical alloying process (MM) in nanocrystalline materials produced by the high-energy ball-milling synthesis method (wherein a magnet is placed close to the cell to apply a strong magnetic pulling force on the magnetic milling balls), the impact energy is usually 1000 times higher than that of CM materials [64]. Mechanical contamination can be attributed to the milling tools, but utilizing optimal milling speeds and times may successfully decrease contamination [65]. In this work, such contamination was reduced by sealing the vial with a flexible ring after the powder was loaded. However, the high-energy ball-milling method has additional drawbacks besides contamination, including a long milling time, the presence of agglomerates [45], high temperature [66], the easy oxidation of powder, and residual strain in the crystallized phase. The significant effects of these parameters may therefore explain the better performance of the CM samples in comparison to the MM samples. However, all samples improved in terms of reflection loss, with all of them exhibiting more than 90% absorption.

Despite these results, the EM wave absorption characteristics of magnetite at varying sizes remain inadequate, the preparation trajectory of magnetite material (extracted from milled scale waste) and ball-milling-adjustable adsorption properties are still motivating research questions. Ameliorations in the electromagnetic wave absorption performance of magnetite at various scales, as well as recombination with other materials, deserve further study [49].

### 3.3. Electromagnetic Properties

#### 3.3.1. Complex Permittivity and Complex Permeability

The complex permittivity ($\varepsilon_{r}$′–$j\varepsilon_{r}$″), complex permeability (μ′–jμ″), and microwave absorption characteristics of magnetite composites produced at various milling durations and thicknesses of 1 and 2 mm were examined in the X and Ku bands (8–18 GHz) frequency ranges. The real components (*ε*′ and *μ*′) represent the storage capacity of electric and magnetic energies, respectively, whereas the imaginary parts (*ε*″ and *μ*″) indicate the loss capacity of electric and magnetic energies, respectively [67,68].

**Figure 7:**
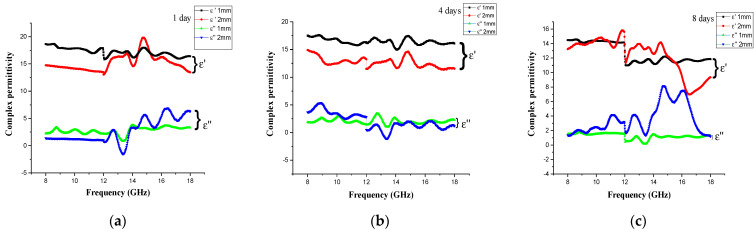

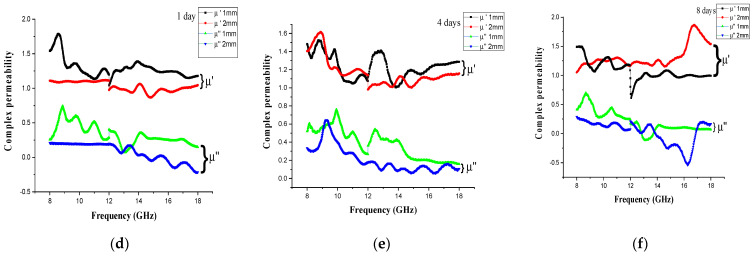
The frequency-dependent (**a**–**c**) complex permittivity and (**d**–**f**) complex permeability of the Fe_3_O_4_/CM at 1, 4, and 8 days of milling time at 1 and 2 mm thicknesses.

Figure 7 shows the spectra of the Fe_3_O_4_/CM absorption material’s complex permittivity (ε) and complex permeability (μ) as a function of frequency for various milling times. Curve of the real *ε*′ and imaginary *ε*″ parts of the complex permittivity of the absorption material as a function of frequency are shown in Figure 7a–c. As can be observed in the graphs, for all milled samples (1, 4, and 8 days), the ε′ spectra show meager variations and some wavy behavior in the entire frequency range utilized in this work. However, the ε′ values decreased from ~16 to ~11.2 as the milling time increased from 4 to 8 days at a 1 mm thickness, whereas the *ε*″ values of all milled samples at a 1 mm thickness showed a constant trend versus the changing frequency at the three different milling times. For the 2 mm samples, the imaginary portion (ε″) of complex permittivity throughout the 8–18 GHz range showed significant fluctuation at high frequencies, which might have been due to charge polarization. Figure 7d–f shows the complex permeability (μ′ and μ″) values of the Fe_3_O_4_/CM absorption material at 1, 4, and 8 days of milling time and thicknesses of 1 and 2 mm. The real (μ′) complex permeability decreased with increases of frequency until it approached 1. The values of μ′ and μ″ of Fe_3_O_4_/CM of 1 and 4 days of milling were roughly around 1 and 0, respectively. Throughout the observed frequency range for a 2 mm sample, the real relative permeability (μ′) for an 8 day sample varied more (μ′ ~1.5) and the imaginary relative permeability (μ″) remains constant (μ″ ~3). As the μ′ and *µ*″ values of all samples remained almost constant over the studied frequency range, these results indicate that varying the milling time did not contribute to the magnetic loss. 

**Figure 8:**
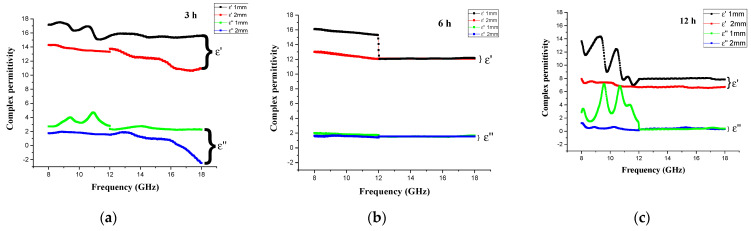

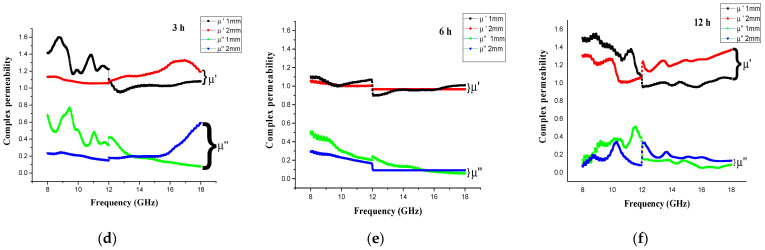
The frequency-dependent of (**a**–**c**) complex permittivity and (**d**–**f**) complex permeability of the Fe_3_O_4_/MM at 3, 6, and 12 h of milling at 1 and 2 mm thicknesses.

The complex permittivity and permeabilities of the Fe_3_O_4_/MM composites measured after 3, 6, and 12 h of milling between 8 and 18 GHz are shown in Figure 8. The real part was highest when the milling time was 3 h, and as the milling time increased to 6 and 12 h, the real part began to decrease in the frequency range of 8–12 GHz before remaining essentially unaltered at higher frequencies (12–18 GHz). The imaginary permittivity of all composites decreased to roughly 1 as the particle size became smaller, though the ε″ of the 12 h milling time sample with a thickness of 1 mm was a little bit higher than that of the 3 and 6 h samples in the beginning but stayed the same at above 12 GHz, which may have been caused by the distribution of particles. As shown in Figure 8c, broad peaks of ε′ and ε″ for the samples milled for 12 h in the frequency range of 8–12 GHz were observed at the thickness of 1 mm. However, broad peaks were not observed within this frequency range for the sample milled for 6 h. In general, all samples showed higher values of ε′ and ε″ at low frequencies due to the low resistivity of magnetite, thus leading to the generation of electric charge transference.

Figure 8d–f shows the spectra of complex permeability vs frequency obtained from our samples, with maximum values of 1.3 and 0.6 for μ′ and μ″, respectively, after 3 h of milling at a 2 mm thickness. Even after milling for 12 h, the sample showed a similar pattern with a minor hump at 12 GHz. The sample milled for 6 h showed almost stable μ′ values over the studied frequency range.

Generally, the ε′ of the extracted Fe_3_O_4_ particles from mill scale decreased as the frequency rose. This trend is diffuse with ferrite and can explained by the Maxwell–Wenger polarization model [44]. In addition, the ε″ values also appeared to decrease with frequency. In comparison, the samples milled for 8 days at a 2 mm thickness and the sample milled for 3 h at a 1 mm thickness showed the best microwave absorber performance among the CM samples and MM samples, respectively. In contrast, the 3 h Fe_3_O_4_/MM sample had higher ε′ and ε″ values than the 8 day Fe_3_O_4_/CM sample throughout the frequency range of 8–18 GHz. The ε′ of the 3 h Fe_3_O_4_/MM sample varied from 17.5 to 16.03, while the ε″ varied between 3 and 2.8. The ε′ values ranged from 13.2 to 9.8 for the 8 d Fe_3_O_4_/CM sample, whereas the ε″ values varied between 1.7 and 1.9. These findings reveal that the complex permittivity of the manufactured samples was considerably enhanced when particle sizes were reduced from the large to small scale utilizing the milling process. It can be concluded that a high ε′ value is beneficial for increasing electromagnetic impedance matching and improving magnetic-particle microwave absorption performance.

The complex permittivity and permeability spectra of some prepared composites revealed negative permittivity or permeability. These negative values may be attributed to the strong magnetic field induced by a large eddy current that could have cancelled or dominated the external magnetic field. It is well-known that in magnetic metal-insulator materials, the magnetic loss is mostly associated with eddy current loss and magnetic resonance, which is composed of natural resonance and domain wall resonance [69]. In addition, there are natural materials that exhibit negative permittivity or permeability at certain frequency ranges, including ferrimagnetic materials at microwave frequencies (high-frequency range, i.e., 12–18 GHz) [70,71,72]. The negative imaginary parts of complex permittivity or permeability have been observed in many composite systems, such as multiwalled carbon nanotube composites [73], porous Fe_3_O_4_/SnO_2_ core/shell nanorods [74], FeCo/C/Fe_2.5_Cr_0.5_Se_4_ nanocomposites [75], and SiC nanowires [76]. Notably, the ε′ and μ″ results of some of our composites were negative over the higher test frequency ranges, indicating that the magnetic component of the composites had no effect. The magnetic energy radiated out instead of being absorbed in these samples, which may have been linked to the creation of resonances and eddy current effects in contrast to the magnetic loss capacity of positive ε″ and μ″.

#### 3.3.2. Loss Tangent

The dielectric loss tangent (tan$\delta_{\varepsilon }$) and the magnetic loss tangent (tan$\delta_{\mu }$) indicate how materials damper electromagnetic waves through electrical and magnetic impacts, respectively [77]. 

The following equation expresses the dielectric loss:

(6) $$tan\delta_{\varepsilon }=\frac{\varepsilon^{\prime \prime }}{\varepsilon^{\prime }}$$

The magnetic loss is expressed by Equation (7):

(7) $$tan\delta_{\mu }=\frac{\mu^{\prime \prime }}{\mu^{\prime }}$$

The electromagnetic wave loss factor (*tan*δ), which is a standard for evaluating the contributions of dielectric and magnetic losses, is usually used to assess a material’s absorption capacity, and it can be described as [78]:

(8) $$tan\delta =tan\delta_{\varepsilon }+tan\delta_{\mu }$$

**Figure 9:**
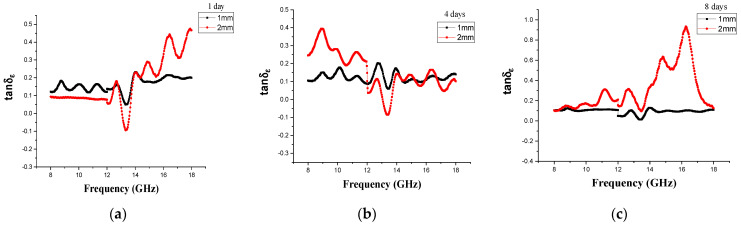

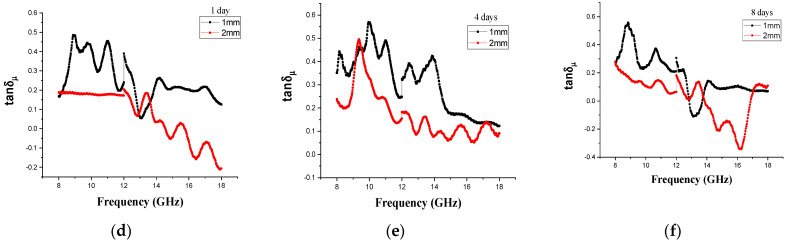
(**a**–**c**) Electric loss tangents and (**d**–**f**) magnetic loss tangents of the Fe_3_O_4_/CM milled for (I) 1 day, (II) 4 days, and (III) 8 days at 1 and 2 mm thicknesses.

**Figure 10:**
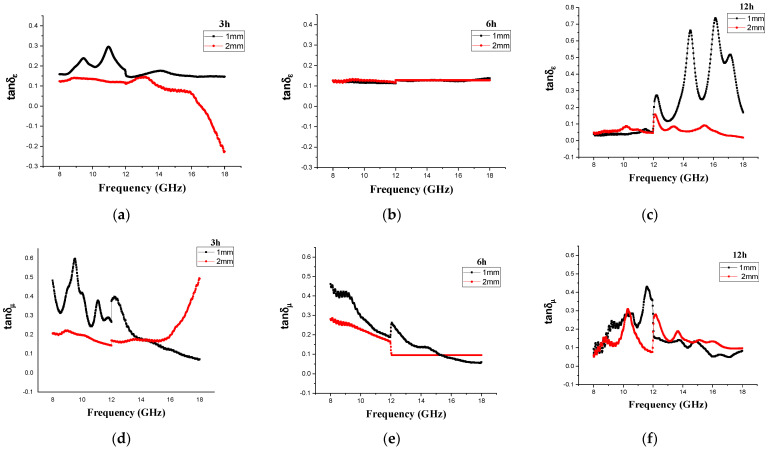
(**a**–**c**) Electric loss tangents and (**d**–**f**) magnetic loss tangents of the Fe_3_O_4_/MM at (I) 3 h, (II) 6 h, and (III) 12 h of milling at 1 and 2 mm thicknesses.

Microwave loss capability can be represented by the loss tangent. Figure 9 and Figure 10 illustrate the dielectric and magnetic loss tangents, respectively, of the CM and MM samples at three different milling times in the 8–18 GHz range. The dielectric loss tangent of the Fe_3_O_4_/CM absorption material is plotted as a function of frequency in Figure 9. The results showed that the increasing milling time intensified dielectric loss by tuning the dielectric coefficients [78]. In accordance with absorbing property explanations, Figure 6, and the values of the reflection losses (RLs), it was reconfirmed that the sample milled for 8 days with a 2 mm thickness showed better performance than the other two samples in terms of microwave absorption. In other words, this sample displayed the maximum absorption frequency width with a thin thickness the maximum effective bandwidth (bandwidth at <−10 dB). Based on the curve in Figure 10c,f, this efficiency in performance could be explained by the fact that the dielectric coefficients changed so that the dielectric loss value in the 8 d sample was higher than that of other samples in the range of 13.5–18 GHz. Thus, it was confirmed that the 8 d sample is a great candidate for microwave absorption studies. The magnetic loss, of which *tan*$\delta_{\mu }$ is a criterion, was almost equal in samples milled for 1 and 4 days and somewhat higher in the sample milled for 8 days (see Figure 9f). In addition. as found from Figure 7a–c, the imaginary component of the complex permittivity initially declined with increasing ball-milling time and then stayed almost constant (~zero) for all samples. As long as the *ε*″ value was near zero, the sample had an extremely low dielectric loss tangent.

Furthermore, the dielectric loss values of the 3 h Fe_3_O_4_/MM composites were significantly greater than those of samples milled for 6 h, which stayed unaltered. Moreover, as shown in Figure 10a,d, the magnetic loss tangent of the 3 h sample was larger than the dielectric loss tangent, indicating that microwave absorption was mostly due to the magnetic effect. Magnetic loss has a very significant effect on the absorption of electromagnetic waves, as electromagnetic energy is converted into heat energy with magnetic loss to enhance the absorption of electromagnetic waves [79]. However, here, the dielectric loss followed an increasing trajectory for the 12 h milled sample in the frequency range above 12 GHz and then again suffered a significant decrease. The influence of different ball-milling times on the magnetic loss tangent of the Fe_3_O_4_/MM absorption material was not considerable, indicating that even though the Fe_3_O_4_/MM absorbent material was milled at different times from large to fine particles and the structure of the material dramatically changed, there was no significant effect on its magnetic loss tangent. It can be concluded that altering the microstructure of an Fe_3_O_4_ absorption material via high-energy ball milling has a significant impact on the material’s dielectric characteristics.

The findings of the electromagnetic parameter and loss factor studies revealed that the ball-milling process had an impact on the microstructure of the Fe_3_O_4_ absorber and that the size of the refiner had a significant effect on its dielectric loss. At increased ball-milling times, the sample possessed smaller grain sizes. Because of different particle sizes caused by milling, defects and voids were produced by lattice deformation, leading to an inconsistency of charges, which changed the capacitance of the samples. Therefore, these defects play an important role in increasing the grain boundaries of milled samples and thus promoting space charge polarization, which leads to grains with high conductivity being separated by grain borders with low conductivity [79]. Space charge polarization leads to changes in the dielectric loss, which depends on the capacitance of samples [80]. As previously stated, we found that the dielectric constant value dropped as milling time increased. Particle sizes are increased as the milling process goes on, so it is entirely possible for these ions to be polarized to an extreme, leading to an increase in the dielectric loss. A large increases in milling time (e.g., 12 h) may have led to decreases in the dielectric constant and dielectric loss, thus degrading the interface polarization for the Fe_3_O_4_ samples. However, the best milling time was found to be 8 d because even though the structural particles were fractured due to sufficient milling time, the fragmented particles were milled into finer and more uniform micron-sized particles. At the shorter milling time, the conductivity and dielectric constant were improved, especially in the high frequency band.

#### 3.3.3. Cole–Cole

**Figure 11:**
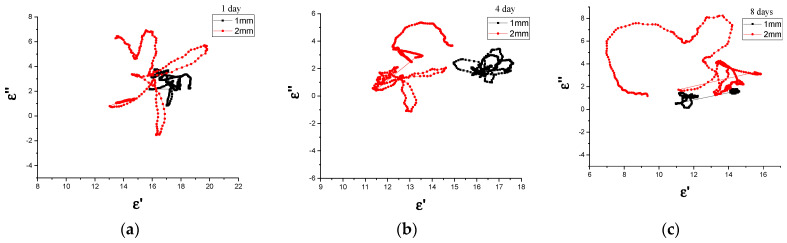
Cole–Cole diagram of the Fe_3_O_4_/CM milled for (**a**) 1 day, (**b**) 4 days, and (**c**) 8 days at 1 and 2 mm thicknesses.

**Figure 12:**
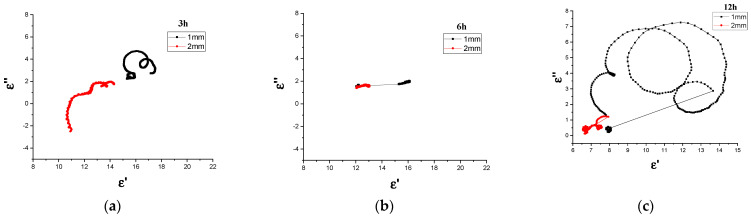
Cole-Cole diagram of the Fe_3_O_4_/MM milled for (**a**) 3 h, (**b**) 6 h, and (**c**) 12 h at 1 and 2 mm thicknesses.

For a system that deviates from its original equilibrium position to return to its original equilibrium state, the buildup of polarization takes a finite time interval before the polarization attains its maximum value [13,81]. The generic name for this phenomenon is dielectric relaxation, where dielectric relaxation has a substantial impact on the electromagnetic absorption characteristics of magnetic materials [82]. To further explain the dielectric relaxation process in detail and examine the dielectric loss mechanism of Fe_3_O_4_, the Debye dipolar relaxation curves of the CM and MM Fe_3_O_4_ absorption materials at different ball-milling periods are displayed in Figure 11 and Figure 12, respectively. As explained above, the relaxation process (multiple semicircle) caused the defects of milled samples. The relationship between ε′ and ε ″ according to the Debye dipolar relaxation model can be expressed as Equation (9) [83]:

(9) $$\varepsilon_{\tau \;}=\varepsilon_{\infty }+\frac{\varepsilon_{s}-\varepsilon_{\infty }\;}{1+j2\pi ƒ\tau }=\varepsilon^{\prime }\;\left(ƒ\right)+i\varepsilon^{\prime \prime }\left(ƒ\right)$$

where $\varepsilon_{s}$ is the static permittivity, $\varepsilon_{\infty }\;$is the dielectric permittivity at the high-frequency limit of EMW (ƒ), as determined by Equations (10) and (11), and τ is polarization relaxation time.

(10) $$\varepsilon^{\prime }\;\left(ƒ\right)=\varepsilon_{\infty }+\frac{\varepsilon_{s}-\varepsilon_{\infty }}{1+{\left(2\pi ƒ\right)}^{2}\;\tau^{2}}$$

(11) $$\varepsilon^{\prime \prime }\;\left(ƒ\right)=\frac{2\pi ƒ\tau \;\left(\varepsilon_{s}-\varepsilon_{\infty }\right)}{1+{\left(2\pi ƒ\right)}^{2}\;\tau^{2}}$$

Based on Equations (8), and (9), the correlation between ε′ and ε″ can be obtained with Equation (12) [84]:

(12) $${\left(\;\varepsilon^{\prime }-\frac{\varepsilon_{s}-\varepsilon_{\infty }}{2\;}\right)}^{2}+{\left(\varepsilon^{\prime \prime }\right)}^{2}={\left(\frac{\varepsilon_{s\;}-\varepsilon_{\infty }}{2}\right)}^{2}$$

Therefore, the spectrum of ε′ versus ε″ would be a single semicircle called a Cole–Cole semicircle. Each semicircle represents a Debye dipolar relaxation [85]. Figure 11 and Figure 12 show the ε′ values plotted as functions of the corresponding ε″ values for CM/ and MM/Fe_3_O_4_ absorption materials at different milling times in the X and Ku bands of 8–18 GHz, demonstrating the presence of dielectric relaxation in these samples. The Cole–Cole curve of the 1 and 4 day Fe_3_O_4_ CM composites show quite a few semi-circles. This finding may be ascribed to the large crystallite size of the Fe_3_O_4_ microparticles. However, multi-arcs for 8 day Fe_3_O_4_/CM can be observed, which may signify the presence of more than one relaxation process of the electrical insulation. Multiple relaxations could have been terminated due to interface polarization, which may have been due to the inhomogeneous interfaces of magnetic microparticles. Figure 12 shows that the 3 h Fe_3_O_4_/MM samples with both 1 and 2 mm thicknesses obtained two overlapping semicircles, which clarified the double dielectric relaxation process in the 3 h e_3_O_4_/MM sample, while there were no obvious arcs with increasing frequency for 6 h Fe_3_O_4_/MM. It is obvious that the 1 mm samples milled for 12 h displayed more semicircles in comparison to others, suggesting that the long milling time endowed Fe_3_O_4_ with multiple dielectric relaxation processes. Each semicircle is associated with Debye relaxation, which could have effectively enhanced the dielectric loss and improved the electromagnetic absorption of Fe_3_O_4_.

## 4. Conclusions

Magnetite composites with different particle sizes were successfully extracted from mill scale waste products via a mechanical activation process at three milling durations. In the frequency range of 8–18 GHz, the influence of ball-milling time on the microstructure and absorption characteristics of Fe_3_O_4_/CM and Fe_3_O_4_/MM was examined. The XRD results indicated that the CM and MM samples had the same cubic and monoclinic crystal structure phases. The complex permittivity and permeability, as well as their correlation to microwave absorption characteristics, were studied. The Fe_3_O_4_/CM sample milled for 8 days displayed the optimum microwave absorption properties, with a minimum RL of −30.83 dB at 9.72 GHz and a matching thickness of 2.0 mm. The corresponding RL bandwidth of less than −10 dB was 2.30 GHz. Meanwhile, the Fe_3_O_4_ with a smaller particle size (after 3 h of milling) exhibited more optimum microwave absorption properties of −20.59 dB at a high frequency band (16.35 GHz) and thin thickness (1 mm) than the corresponding larger ones in the microscale due to improved impedance matching. As a result, the Fe_3_O_4_/CM and Fe_3_O_4_/MM composite achieved excellent electromagnetic absorption properties and could sufficiently meet lightweight, thin thickness, strong absorptive capacity, and broad absorption band requirements. In summary, the authors of this work have presented insights into effective low-cost products derived from mill scale waste via the ball-milling method, their performance as microwave absorption materials (MAMs), their electromagnetic properties.

## Figures and Tables

**Table 1:** The lattice parameters of Fe_3_O_4_/CM with milling times of 1, 4, and 8 days and Fe_3_O_4_/MM at high-energy ball milling (HEBM) times of 3, 6, and 12 h.

Samples	Milling Time	Compound	Chemical Formula	JCPDS Ref	Crystal System	Space Group	Lattice Parameter			The Ratios of Compounds (W%)	Unit Cell Volume, V_cell_	
							*a*(Å)	*b*(Å)	*c*(Å)			Crystallinity (%)
Fe_3_O_4_/CM	1 day	Magnetite (Tri-iron Tetraoxide)	Fe_3_O_4_	98-004-1157	Cubic	F d-3 m	8.4050	8.4050	8.4050	45.7	593.76	79
		Magnetite (Iron Diiron(III) Oxide-Hp)	Fe_3_O_4_	98-005-2579	Orthorhombic	P b c m	2.7990	9.4100	9.4830	0.0	249.77	
		Wustite	FeO	98-008-8082	Cubic	F m-3 m	4.2900	4.2900	4.2900	54.3	78.95	
	4 days	Magnetite	Fe_3_O_4_	98-004-1424	Cubic	F d-3 m	8.4910	8.4910	8.4910	100.0	612.18	76.42
	8 days	Magnetite	Fe_3_O_4_	98-004-1424	Cubic	F d-3 m	8.4910	8.4910	8.4910	100.0	612.18	80.16
Fe_3_O_4_/MM	3 h	Magnetite	Fe_3_O_4_	98-007-7864	Monoclinic	P 1 2/c 1	5.9440	5.9250	16.7750	100.0	590.78	83.93
	6 h	Magnetite	Fe_3_O_4_	98-007-7864	Monoclinic	P 1 2/c 1	5.9440	5.9250	16.7750	100.0	590.78	88.31
	12 h	Magnetite	Fe_3_O_4_	98-007-7864	Monoclinic	P 1 2/c 1	5.9440	5.9250	16.7750	100.0	590.78	86.46

**Table 2:** The milling time, 2θ of the highest intensity (2θ_h_), FWHM, intensity, and average crystallite size of Fe_3_O_4_ at various milling times.

Sample	Milling Time	2*θ*_h_	FWHM (^o^)	Intensity	Crystallite Size, D (nm)
Fe_3_O_4_/CM	1 day	35.41	0.3507	2414.37	24
	4 days	35.35	0.76155	604.16	11
	8 days	35.45	0.70721	1238.79	12
Fe_3_O_4_/MM	3 h	35.38	1.65391	662.12	5.1
	6 h	35.37	1.67468	930.91	4.9
	12 h	35.39	0.87238	924.55	9.6

**Table 3:** Microwave absorption properties and particle sizes of magnetite prepared via CM and MM methods with milling times of 1, 4, and 8 days and 3, 6, and 12 h, respectively, at thicknesses of 1 and 2 mm.

Sample	Milling Time	Peak Value, ƒ_m_ (GHz)	Thickness (mm)	Minimum RL Value (dB)	Frequency Bandwidth (GHz) (RL < −10 dB)
Fe_3_O_4/_CM	1 day	16.11	1	−16.44	2.52
		8.66	2	−26.21	1.76
	4 days	15.39	1	−25.96	1.86
		9.26	2	−12.26	0.50
	8 days	12.06	1	−2.48	1.17
		9.72	2	−30.83	2.30
Fe_3_O_4/_MM	3 h	16.35	1	−20.59	2.43
		9.42	2	−16.48	2.26
	6 h	11.96	1	−3.53	1.86
		9.98	2	−18.91	2.48
	12 h	16.74	1	−16.36	0.93
		12.06	2	−10.56	0.21

**Table 4:** The highest reflection loss of microwave absorbing CM and MM magnetite.

Samples	Milling Time	Minimum RL Value (dB)	Frequency Bandwidth (GHz) (RL < −10 dB)	Absorption (%)
Fe_3_O_4_/CM	1 day	−26.21	2.52	99%
	8 days	−30.83	2.30	99.9%
Fe_3_O_4_/MM	3 h	−20.59	2.43	99%
	6 h	−18.91	2.48	90%
